# Crystal structures of two 2,3-diaryl-2,3-di­hydro-4*H*-1,3-benzo­thia­zin-4-ones

**DOI:** 10.1107/S2056989018002049

**Published:** 2018-02-20

**Authors:** Hemant P. Yennawar, Michaela J. Buchwalter, Baylee K. Colburn, Lee J. Silverberg

**Affiliations:** a The Pennsylvania State University, Dept. Biochemistry and Molecular Biology, University Park, PA 16802, USA; b Pennsylvania State University, Schuylkill Campus, 200 University Drive, Schuylkill Haven, PA 17972, USA

**Keywords:** crystal structure, benzo­thia­zinones, envelope pucker

## Abstract

The syntheses and crystal structures of 2,3-bis­[3-(tri­fluoro­meth­yl)phen­yl]-2,3-di­hydro-4*H*-1,3-benzo­thia­zin-4-one (di-*m*-CF_3_; C_22_H_13_F_6_NOS) (**1**) and 2,3-bis­(4-methyl­phen­yl)-2,3-di­hydro-4*H*-1,3-benzo­thia­zin-4-one (di-*p*-CH_3_; C_22_H_19_NOS) (**2**) are reported. Each structure is racemic: the asymmetric unit of **2** consists of two mol­ecules. In both **1** and **2**, the six-membered 1,3-thia­zine ring is close to an envelope conformation with the chiral C atom forming the flap. In **1**, the 2-aryl group is pseudo-equatorial, while in **2** it is pseudo-axial. In **1**, the pendant aryl rings form a V shape with an inter-centroid distance of 3.938 (3) Å and an acute dihedral angle of 48.3 (2)° between them. Both CF_3_ groups are disordered over two orientations in 0.687 (19):0.313 (19) and 0.667 (16):0.33 (16) ratios. In each of the independent mol­ecules of **2**, the aryl rings are almost orthogonal to each other [dihedral angles = 85.50 (12) and 86.07 (11)°]. In both structures, the chiral C atom and the O atoms participate in C—H⋯O-type hydrogen bonding between symmetry-related mol­ecules of **1** or the independent enanti­omers in **2**, forming chains along the *c*-axis direction in **1** and the *b*-axis direction in **2**. Additionally, in **1**, π–π contacts of both face-to-face and edge-to-face type, as well as π-H⋯O and π-H⋯F inter­actions are observed. In **2**, a racemic mixture of mol­ecules forms layers in the *ac* plane linked by weak π–π and C—H⋯π inter­actions.

## Abstract 2

Crystal structures of two benzo­thia­zinones belonging to a class of compounds implicated in anti­microbial, anti­tumour, and HIV-RT inhibitory activity are reported.

## Chemical context   

*N*-Aryl (*R* ^1^ = aryl or heteroar­yl) 2,3-di­hydro-4*H*-1,3-benzo­thia­zin-4-ones display anti­tumor (Feng *et al.*, 2015 ▸; Kamel *et al.*, 2010 ▸; Nofal *et al.*, 2014 ▸) and anti­microbial (Mandour *et al.*, 2007 ▸) activity, as well as inhibition of HIV-RT (Jeng *et al.*, 2015 ▸), and cyclo­oxygenase COX-2 enzyme (Zarghi *et al.*, 2009 ▸).
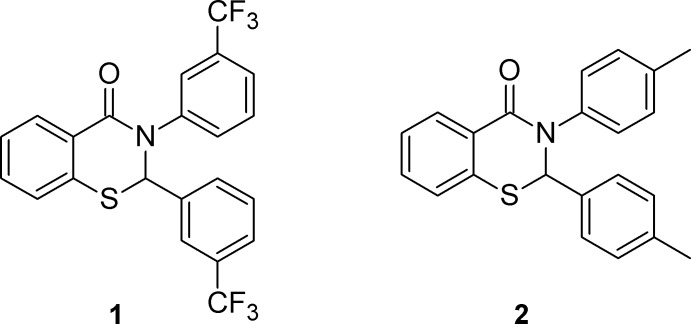


As part of our studies in this area, we have previously reported the crystal structures of a number of 2-aryl-3-phenyl- and 3-aryl-2-phenyl-2,3-di­hydro-4*H*-1,3-benzo­thia­zin-4-ones (Yennawar *et al.*, 2013 ▸, 2014 ▸, 2015 ▸, 2016 ▸). Herein we report the syntheses and crystal structures of two 2,3-diaryl-2,3-di­hydro-4*H*-1,3-benzo­thia­zin-4-ones (di-*m*-CF_3_ **1**, and di-*p*-CH_3_ **2**). Each has been synthesized using the same T3P/pyridine method that was used for the previously reported compounds.

## Structural commentary   

**Figure 1:**
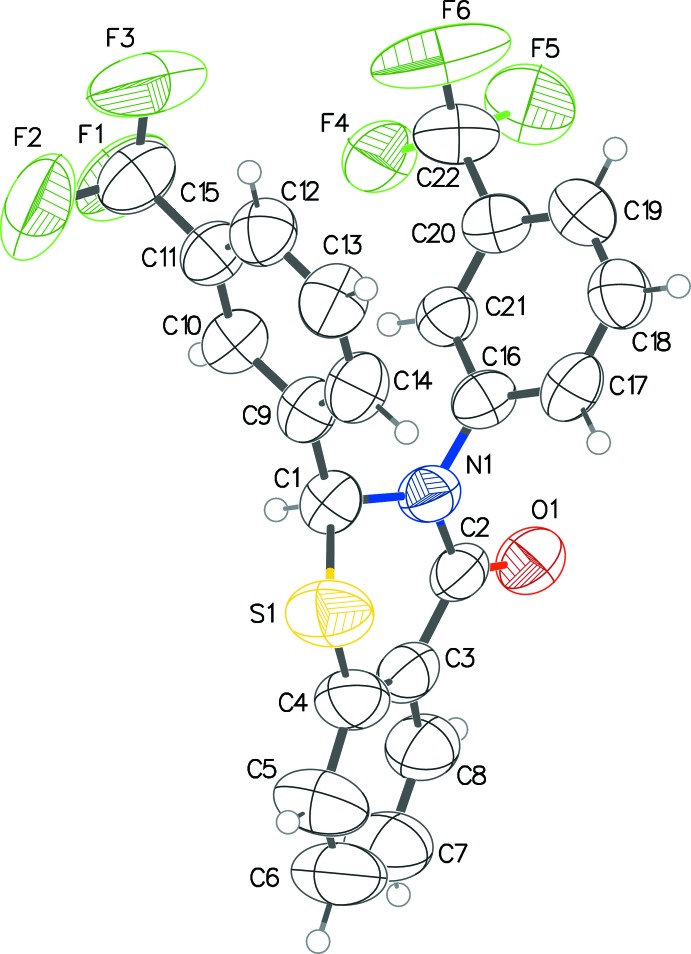
The mol­ecular structure of **1** with displacement ellipsoids drawn at the 50% probability level. Disorder in the CF_3_ groups is not shown for clarity.

The title compounds are shown in Figs. 1 ▸ and 2 ▸. In **1**, the 2-aryl group is pseudo-equatorial, unlike the structures that we have reported previously, while in **2** it is pseudo-axial (both independent mol­ecules) (Yennawar *et al.*, 2013 ▸, 2014 ▸, 2015 ▸, 2016 ▸). The benzo­thia­zine rings in both **1** and **2** have envelope conformations with the chiral carbon atom forming the flap, with puckering parameters in **1** of *Q* = 0.596 (7) Å, θ = 118.2 (8)°, φ = 22.7 (9)° and in **2** (mol­ecules *A* and *B*) *Q* = 0.5490 (19) and 0.5715 (17) Å; θ = 63.5 (2) and 116.31 (19)°; φ = 40.8 (2) and 223.5 (2)°, respectively. In **1**, the pendant aryl rings form an approximate V shape with an acute dihedral angle of 48.3 (2)° and inter-centroid distance of 3.938 (3) Å between them. In each of the independent mol­ecules of **2**, the aryl rings are almost orthogonal to each other [dihedral angles = 85.50 (12) for the C1 mol­ecule and 86.07 (11)° for the C23 mol­ecule].

## Supra­molecular features   

**Figure 3:**
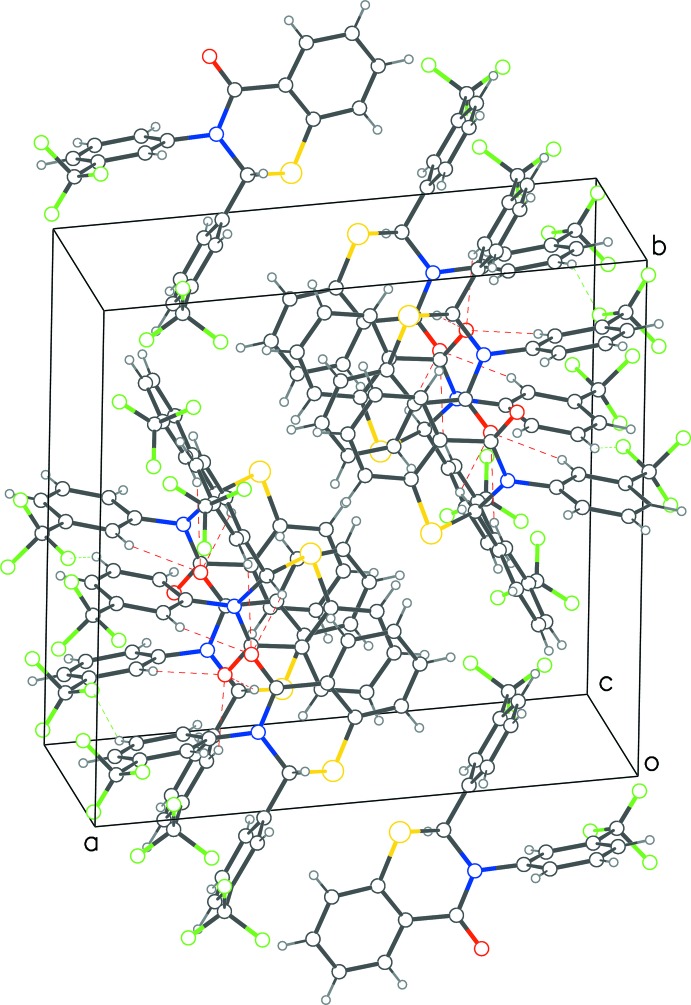
Packing diagram of **1** with red dotted lines representing C—H⋯O and green dotted lines showing C(π)—H⋯F contacts forming chains comprising of alternating enanti­omers, along the *c*-axis direction.

**Figure 4:**
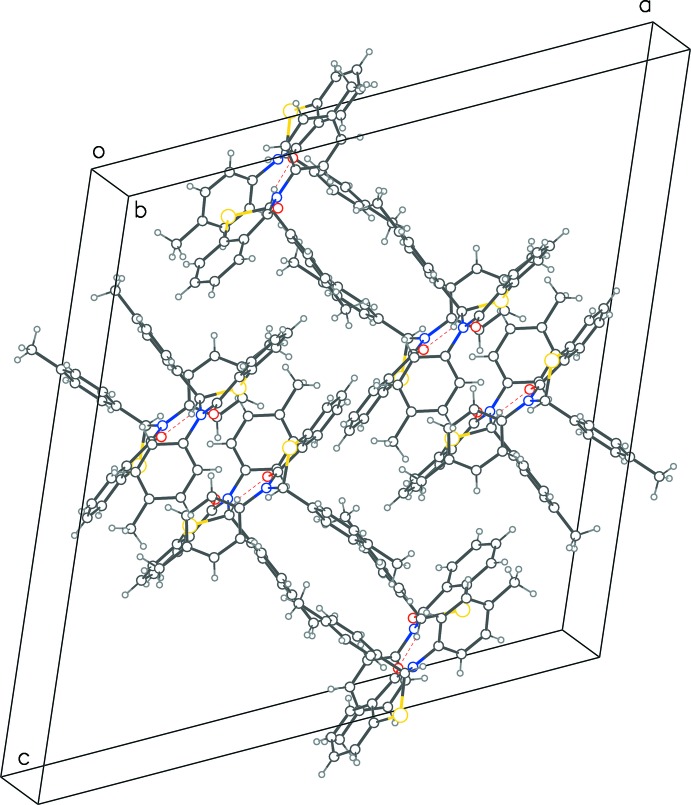
Packing diagram of **2** with red dotted lines representing C—H⋯O contacts forming chains along the *b*-axis direction comprising of alternating enanti­omers.

In the two structures, C—H⋯O inter­actions (Tables 1 ▸ and 2 ▸) in which the chiral carbon atom (C1 in **1**; C1 and C23 in **2**) donates its H atom to the oxygen atom of a symmetry related mol­ecule in **1**, or the independent enanti­omer in **2**. This results in infinite chains along the *c*- and *b*-axis directions, respectively. In **1**, these chains are further consolidated by π-H⋯O and π-H⋯F inter­actions [C10⋯O1^i^ = 3.370 (5); C21⋯O1^i^ = 3.368 (6) Å; symmetry code: (i) *x*, −*y* + 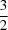, *z* + 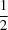], and π-H⋯F [C14⋯F2*A* ^ii^ = 3.27 (2); C18⋯F4^ii^ = 3.394 (10) Å; symmetry code: (ii) *x*, *y*, *z* − 1] (Fig. 3 ▸), although in the latter the participating mol­ecules reverse their donor and acceptor roles. Within these chains, the fused benzene rings of adjacent mol­ecules exibit inter­molecular face-to-face type π–π inter­actions [*Cg*⋯*Cg* = 3.9920 (15) Å]. The structure also features inter­actions of edge-to-face type between the fused benzene and 2-aryl rings [*Cg*⋯*Cg* = 5.0083 (14) Å]. In **2**, weak π–π [*Cg*⋯*Cg* = 4.735 (2) Å] and C—H⋯π inter­actions (Table 2 ▸) are present between the racemic mixture of mol­ecules in *ac* plane (Fig. 4 ▸).

### Database survey   

Along with the structures we have previously published (Yennawar *et al.*, 2013 ▸, 2014 ▸, 2015 ▸, 2016 ▸), crystal structures of three other compounds with the same 2,3-di­hydro-4*H*-1,3-benzo­thia­zin-4-one core have been reported (Kröger *et al.* 2015 ▸; Wang *et al.*, 2017 ▸; Yin *et al.*, 2016 ▸). The structure reported by Yin displays an envelope pucker with the 2-C atom as the flap for the thia­zine ring that is sandwiched between two fused rings. CIFs were not available for the other two compounds.
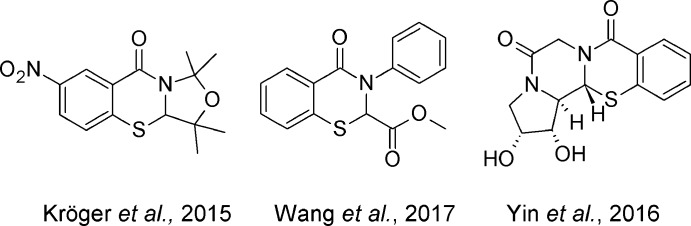


## Synthesis and crystallization   

General: A two-necked 25 ml round-bottom flask was oven-dried, cooled under N_2_, and charged with a stir bar and the imine (6 mmol). Thio­salicylic acid (0.93 g, 6 mmol) and then 2-methyl­tetra­hydro­furan (2.3 mL) were added and the solution was stirred. Pyridine (1.95 mL, 24 mmol) and, finally, 2,4,6-tripropyl-1,3,5,2,4,6-trioxatri­phospho­rinane-2,4,6-trioxide (T3P) in 2-methyl­tetra­hydro­furan (50 weight percent; 7.3 mL, 12 mmol) were added. The reaction was stirred at room temperature and followed by TLC. The mixture was poured into a separatory funnel with di­chloro­methane and distilled water. The layers were separated and the aqueous one was then extracted twice with di­chloro­methane. The organic layers were combined and washed with saturated sodium bicarbonate and then saturated sodium chloride. The organic layer was dried over sodium sulfate and concentrated under vacuum. The crude product was chromatographed on 30 g flash silica gel and then recrystallized.

2,3-Bis[3-(tri­fluoro­meth­yl)phen­yl]-2,3-di­hydro-4*H*-1,3-benzo­thia­zin-4-one (**1**): Recrystallized from 2-propanol solution. Yield: 0.5199 g (19%), m.p. 392–393 K. Colorless blocks of **1** were grown by slow evaporation from cyclo­hexane solution.

2,3-Bis(4-methyl­phen­yl)-2,3-di­hydro-4*H*-1,3-benzo­thia­zin-4-one **2**: Recrystallized from 2-propanol solution. Yield: 0.6288 g (30%), m.p. 412–414 K. Colorless needles of **2** were grown by slow evaporation from ethanol solution.

## Refinement   

Crystal data, data collection and structure refinement details for both compounds are summarized in Table 3 ▸. Both CF_3_ groups of **1** are disordered over two orientations in a 0.687 (19):0.313 (19) ratio for the C15 group and a 0.667 (16):0.33 (16) ratio for the C22 group·The disorder was restrained using SIMU and DELU commands in *SHELX* for the twelve resulting atoms.

## Supplementary Material

Crystal structure: contains datablock(s) 1, 2. DOI: 10.1107/S2056989018002049/hb7726sup1.cif

Structure factors: contains datablock(s) 1. DOI: 10.1107/S2056989018002049/hb77261sup2.hkl

Supporting information file. DOI: 10.1107/S2056989018002049/hb77261sup4.mol

Structure factors: contains datablock(s) 2. DOI: 10.1107/S2056989018002049/hb77262sup3.hkl

Supporting information file. DOI: 10.1107/S2056989018002049/hb77262sup5.mol

Supporting information file. DOI: 10.1107/S2056989018002049/hb77261sup6.cml

Supporting information file. DOI: 10.1107/S2056989018002049/hb77262sup7.cml

CCDC references: 1821702, 1821701

Additional supporting information:  crystallographic information; 3D view; checkCIF report

## Figures and Tables

**Figure 2:**
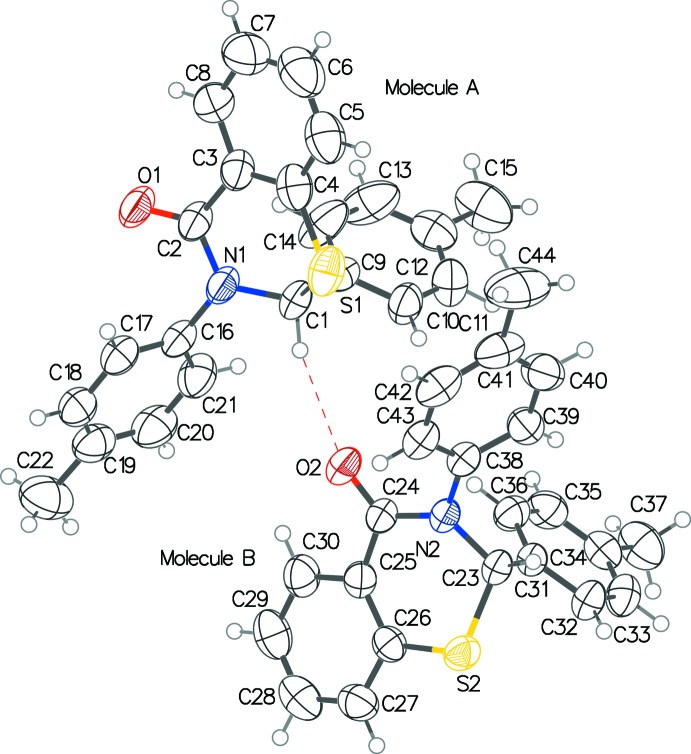
The two independent mol­ecules of **2** showing the C—H⋯O inter­action between the enanti­omers. The displacement ellipsoids are drawn at the 50% probability level.

**Table 1:** Hydrogen-bond geometry (Å, °) for **1** [Chem scheme1]

*D*—H⋯*A*	*D*—H	H⋯*A*	*D*⋯*A*	*D*—H⋯*A*
C1—H1⋯O1^i^	0.98	2.61	3.360 (5)	133
C10—H10⋯O1^i^	0.93	2.59	3.370 (5)	141
C14—H14⋯F2*A* ^ii^	0.93	2.59	3.27 (2)	130
C18—H18⋯F4^ii^	0.93	2.56	3.394 (10)	149
C21—H21⋯O1^i^	0.93	2.50	3.368 (6)	156

Symmetry codes: (i) 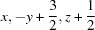; (ii) 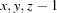.

**Table 2:** Hydrogen-bond geometry (Å, °) for **2** [Chem scheme1] *Cg*4 and *Cg*8 are the centroids of the C16–C21 and C31–C36 rings, respectively.

*D*—H⋯*A*	*D*—H	H⋯*A*	*D*⋯*A*	*D*—H⋯*A*
C1—H1⋯O2	0.98	2.46	3.399 (3)	161
C23—H23⋯O1^i^	0.98	2.34	3.268 (2)	158
C28—H28⋯*Cg*8^ii^	0.93	2.60	3.514 (3)	169
C32—H32⋯*Cg*4	0.93	2.90	3.818 (2)	171

Symmetry codes: (i) 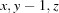; (ii) 
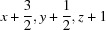
.

**Table 3:** Experimental details

	**1**	**2**
Crystal data		
Chemical formula	C_22_H_13_F_6_NOS	C_22_H_19_NOS
*M*_r_	453.39	345.44
Crystal system, space group	Monoclinic, *P*2_1_/*c*	Monoclinic, *C*2/*c*
Temperature (K)	298	298
*a*, *b*, *c* (Å)	16.602 (6), 15.546 (6), 7.915 (3)	24.821 (7), 12.151 (3), 26.219 (7)
β (°)	99.344 (8)	112.470 (4)
*V* (Å^3^)	2015.8 (13)	7307 (3)
*Z*	4	16
Radiation type	Mo *K*α	Mo *K*α
μ (mm^−1^)	0.23	0.19
Crystal size (mm)	0.10 × 0.03 × 0.02	0.29 × 0.28 × 0.2

Data collection		
Diffractometer	Bruker SMART CCD	Bruker SMART CCD
Absorption correction	Multi-scan (*SADABS*; Bruker, 2001 ▸)	Multi-scan (*SADABS*; Bruker, 2001 ▸)
*T*_min_, *T*_max_	0.738, 0.9	0.100, 0.85
No. of measured, independent and observed [*I* > 2σ(*I*)] reflections	17435, 4882, 1964	26265, 8985, 5453
*R*_int_	0.062	0.046
(sin θ/λ)_max_ (Å^−1^)	0.663	0.668

Refinement		
*R*[*F* ^2^ > 2σ(*F* ^2^)], *wR*(*F* ^2^), *S*	0.111, 0.329, 0.91	0.058, 0.174, 0.96
No. of reflections	4882	8985
No. of parameters	337	455
No. of restraints	72	0
H-atom treatment	H-atom parameters constrained	H-atom parameters constrained
Δρ_max_, Δρ_min_ (e Å^−3^)	0.64, −0.49	0.32, −0.33

Computer programs: *SMART* and *SAINT* (Bruker, 2001 ▸), *SHELXS97* and *SHELXL97* (Sheldrick, 2008 ▸) and *OLEX2* (Dolomanov *et al.*, 2009 ▸).

## supplementary crystallographic information

### 2,3-Bis[3-(trifluoromethyl)phenyl]-2,3-dihydro-4H-1,3-benzothiazin-4-one (1) Crystal data

**Table:**

C_22_H_13_F_6_NOS	*F*(000) = 920
*M**_r_* = 453.39	*D*_x_ = 1.494 Mg m^−^^3^
Monoclinic, *P*2_1_/*c*	Mo *K*α radiation, λ = 0.71073 Å
*a* = 16.602 (6) Å	Cell parameters from 1716 reflections
*b* = 15.546 (6) Å	θ = 2.5–26.2°
*c* = 7.915 (3) Å	µ = 0.23 mm^−^^1^
β = 99.344 (8)°	*T* = 298 K
*V* = 2015.8 (13) Å^3^	Needle, colorless
*Z* = 4	0.10 × 0.03 × 0.02 mm

### 2,3-Bis[3-(trifluoromethyl)phenyl]-2,3-dihydro-4H-1,3-benzothiazin-4-one (1) Data collection

**Table:**

Bruker SMART CCD diffractometer	4882 independent reflections
Radiation source: fine-focus sealed tube	1964 reflections with *I* > 2σ(*I*)
Parallel-graphite monochromator	*R*_int_ = 0.062
phi and ω scans	θ_max_ = 28.1°, θ_min_ = 1.8°
Absorption correction: multi-scan (SADABS; Bruker, 2001)	*h* = −21→18
*T*_min_ = 0.738, *T*_max_ = 0.9	*k* = −20→20
17435 measured reflections	*l* = −10→9

### 2,3-Bis[3-(trifluoromethyl)phenyl]-2,3-dihydro-4H-1,3-benzothiazin-4-one (1) Refinement

**Table:**

Refinement on *F*^2^	Secondary atom site location: difference Fourier map
Least-squares matrix: full	Hydrogen site location: inferred from neighbouring sites
*R*[*F*^2^ > 2σ(*F*^2^)] = 0.111	H-atom parameters constrained
*wR*(*F*^2^) = 0.329	*w* = 1/[σ^2^(*F*_o_^2^) + (0.2*P*)^2^] where *P* = (*F*_o_^2^ + 2*F*_c_^2^)/3
*S* = 0.91	(Δ/σ)_max_ < 0.001
4882 reflections	Δρ_max_ = 0.64 e Å^−^^3^
337 parameters	Δρ_min_ = −0.49 e Å^−^^3^
72 restraints	Extinction correction: SHELXL97 (Sheldrick, 2008), Fc^*^=kFc[1+0.001xFc^2^λ^3^/sin(2θ)]^-1/4^
Primary atom site location: structure-invariant direct methods	Extinction coefficient: 0.118 (13)

### 2,3-Bis[3-(trifluoromethyl)phenyl]-2,3-dihydro-4H-1,3-benzothiazin-4-one (1) Special details

**Table:**

Experimental. The data collection nominally covered a full sphere of reciprocal space by a combination of 4 sets of ω scans each set at different φ and/or 2θ angles and each scan (30 s exposure) covering -0.300° degrees in ω. The crystal to detector distance was 5.82 cm.
Geometry. All esds (except the esd in the dihedral angle between two l.s. planes) are estimated using the full covariance matrix. The cell esds are taken into account individually in the estimation of esds in distances, angles and torsion angles; correlations between esds in cell parameters are only used when they are defined by crystal symmetry. An approximate (isotropic) treatment of cell esds is used for estimating esds involving l.s. planes.
Refinement. Refinement of F^2^ against ALL reflections. The weighted R-factor wR and goodness of fit S are based on F^2^, conventional R-factors R are based on F, with F set to zero for negative F^2^. The threshold expression of F^2^ > 2sigma(F^2^) is used only for calculating R-factors(gt) etc. and is not relevant to the choice of reflections for refinement. R-factors based on F^2^ are statistically about twice as large as those based on F, and R- factors based on ALL data will be even larger. The disorder in the two CF3 moieties were subject to SIMU and DELU Shelx restraints.

### 2,3-Bis[3-(trifluoromethyl)phenyl]-2,3-dihydro-4H-1,3-benzothiazin-4-one (1) Fractional atomic coordinates and isotropic or equivalent isotropic displacement parameters (Å^2^)

**Table:**

	*x*	*y*	*z*	*U*_iso_*/*U*_eq_	Occ. (<1)
S1	0.42945 (8)	0.92091 (9)	0.0814 (2)	0.1041 (7)	
F1	0.1828 (9)	1.0377 (5)	0.6037 (15)	0.126 (4)	0.687 (19)
F1A	0.119 (2)	1.066 (3)	0.498 (4)	0.189 (12)	0.313 (19)
F2	0.2537 (9)	1.1469 (13)	0.6119 (16)	0.177 (6)	0.687 (19)
F2A	0.234 (3)	1.071 (3)	0.628 (3)	0.189 (13)	0.313 (19)
F3	0.1288 (10)	1.1503 (13)	0.4887 (16)	0.180 (7)	0.687 (19)
F3A	0.188 (3)	1.1751 (9)	0.531 (3)	0.150 (11)	0.313 (19)
F4	0.0493 (6)	0.8379 (9)	0.3828 (12)	0.115 (3)	0.667 (16)
F4A	0.023 (2)	0.811 (3)	0.324 (5)	0.203 (14)	0.333 (16)
F5	−0.0427 (5)	0.7991 (8)	0.1878 (11)	0.131 (3)	0.667 (16)
F5A	−0.0572 (10)	0.881 (2)	0.175 (3)	0.151 (11)	0.333 (16)
F6	−0.0248 (11)	0.9333 (7)	0.225 (2)	0.177 (9)	0.667 (16)
F6A	0.0366 (12)	0.9396 (11)	0.329 (2)	0.124 (6)	0.333 (16)
O1	0.2812 (2)	0.6905 (2)	0.0183 (4)	0.0887 (10)	
N1	0.2902 (2)	0.8323 (2)	0.0806 (4)	0.0675 (9)	
C1	0.3401 (3)	0.9014 (3)	0.1739 (6)	0.0669 (11)	
H1	0.3577	0.8816	0.2917	0.080*	
C2	0.3238 (3)	0.7513 (3)	0.0694 (5)	0.0654 (11)	
C3	0.4140 (3)	0.7453 (3)	0.1057 (5)	0.0721 (12)	
C4	0.4660 (3)	0.8163 (4)	0.1067 (7)	0.0897 (14)	
C5	0.5502 (4)	0.8019 (5)	0.1133 (10)	0.127 (2)	
H5	0.5847	0.8485	0.1069	0.152*	
C6	0.5819 (5)	0.7210 (7)	0.1290 (10)	0.143 (3)	
H6	0.6377	0.7128	0.1333	0.172*	
C7	0.5317 (5)	0.6513 (6)	0.1385 (10)	0.131 (2)	
H7	0.5537	0.5963	0.1530	0.157*	
C8	0.4488 (4)	0.6633 (4)	0.1265 (7)	0.0991 (17)	
H8	0.4153	0.6158	0.1324	0.119*	
C9	0.2939 (3)	0.9826 (2)	0.1814 (5)	0.0663 (11)	
C10	0.2680 (3)	1.0050 (3)	0.3319 (6)	0.0705 (11)	
H10	0.2804	0.9698	0.4276	0.085*	
C11	0.2233 (3)	1.0795 (3)	0.3417 (6)	0.0715 (12)	
C12	0.2041 (3)	1.1332 (3)	0.2009 (7)	0.0801 (13)	
H12	0.1730	1.1825	0.2077	0.096*	
C13	0.2313 (3)	1.1127 (3)	0.0526 (6)	0.0848 (14)	
H13	0.2207	1.1494	−0.0412	0.102*	
C14	0.2745 (3)	1.0380 (3)	0.0413 (6)	0.0782 (13)	
H14	0.2912	1.0239	−0.0619	0.094*	
C15	0.1930 (5)	1.1029 (4)	0.5032 (9)	0.0955 (17)	
C16	0.2051 (3)	0.8448 (2)	0.0275 (5)	0.0658 (11)	
C17	0.1733 (3)	0.8600 (3)	−0.1429 (6)	0.0771 (13)	
H17	0.2073	0.8583	−0.2252	0.092*	
C18	0.0922 (3)	0.8776 (3)	−0.1905 (6)	0.0836 (13)	
H18	0.0716	0.8886	−0.3048	0.100*	
C19	0.0414 (3)	0.8791 (3)	−0.0713 (6)	0.0815 (13)	
H19	−0.0137	0.8918	−0.1046	0.098*	
C20	0.0712 (3)	0.8618 (3)	0.0984 (6)	0.0714 (12)	
C21	0.1529 (3)	0.8443 (2)	0.1492 (5)	0.0652 (10)	
H21	0.1730	0.8323	0.2634	0.078*	
C22	0.0157 (4)	0.8648 (4)	0.2259 (9)	0.0948 (17)	

### 2,3-Bis[3-(trifluoromethyl)phenyl]-2,3-dihydro-4H-1,3-benzothiazin-4-one (1) Atomic displacement parameters (Å^2^)

**Table:**

	*U*^11^	*U*^22^	*U*^33^	*U*^12^	*U*^13^	*U*^23^
S1	0.0821 (10)	0.0974 (11)	0.1418 (15)	−0.0087 (7)	0.0449 (9)	0.0044 (8)
F1	0.190 (10)	0.102 (5)	0.105 (6)	0.008 (5)	0.087 (7)	0.019 (4)
F1A	0.18 (2)	0.24 (3)	0.17 (2)	−0.03 (2)	0.109 (17)	−0.02 (2)
F2	0.204 (10)	0.221 (13)	0.120 (7)	−0.074 (10)	0.069 (6)	−0.082 (8)
F2A	0.30 (4)	0.20 (3)	0.077 (11)	0.03 (3)	0.071 (18)	0.009 (16)
F3	0.180 (11)	0.231 (16)	0.151 (8)	0.115 (11)	0.095 (8)	0.052 (9)
F3A	0.26 (3)	0.062 (8)	0.151 (18)	0.008 (14)	0.09 (2)	−0.034 (8)
F4	0.107 (5)	0.182 (9)	0.064 (4)	0.017 (4)	0.034 (3)	0.003 (4)
F4A	0.21 (3)	0.23 (3)	0.19 (3)	0.05 (2)	0.09 (2)	0.09 (2)
F5	0.097 (5)	0.153 (8)	0.151 (6)	−0.041 (5)	0.047 (4)	0.000 (5)
F5A	0.071 (9)	0.23 (3)	0.159 (12)	0.029 (14)	0.043 (8)	0.01 (2)
F6	0.196 (17)	0.114 (6)	0.260 (18)	0.079 (8)	0.160 (15)	0.059 (8)
F6A	0.111 (11)	0.154 (13)	0.119 (10)	0.016 (9)	0.051 (8)	−0.026 (8)
O1	0.095 (2)	0.0711 (19)	0.100 (3)	0.0082 (17)	0.0163 (19)	−0.0159 (17)
N1	0.065 (2)	0.064 (2)	0.076 (2)	0.0045 (16)	0.0163 (17)	−0.0079 (16)
C1	0.075 (3)	0.061 (2)	0.067 (3)	−0.0041 (19)	0.017 (2)	−0.0013 (19)
C2	0.074 (3)	0.062 (2)	0.062 (3)	0.009 (2)	0.016 (2)	−0.0102 (19)
C3	0.080 (3)	0.077 (3)	0.063 (3)	0.008 (2)	0.021 (2)	−0.005 (2)
C4	0.071 (3)	0.114 (4)	0.089 (4)	0.007 (3)	0.028 (2)	0.003 (3)
C5	0.073 (4)	0.142 (6)	0.169 (7)	0.007 (4)	0.029 (4)	0.008 (5)
C6	0.089 (5)	0.190 (9)	0.154 (7)	0.043 (6)	0.031 (4)	−0.004 (6)
C7	0.117 (6)	0.137 (6)	0.135 (6)	0.054 (5)	0.011 (4)	0.004 (5)
C8	0.102 (4)	0.104 (4)	0.089 (4)	0.037 (3)	0.008 (3)	0.003 (3)
C9	0.079 (3)	0.055 (2)	0.066 (3)	−0.0081 (19)	0.017 (2)	−0.0027 (18)
C10	0.080 (3)	0.063 (2)	0.072 (3)	−0.002 (2)	0.023 (2)	0.001 (2)
C11	0.084 (3)	0.062 (2)	0.071 (3)	0.003 (2)	0.021 (2)	0.000 (2)
C12	0.089 (3)	0.065 (3)	0.086 (3)	0.002 (2)	0.013 (2)	−0.003 (2)
C13	0.109 (4)	0.069 (3)	0.076 (3)	0.008 (3)	0.011 (3)	0.009 (2)
C14	0.095 (3)	0.080 (3)	0.061 (3)	−0.003 (2)	0.019 (2)	0.003 (2)
C15	0.123 (5)	0.086 (4)	0.085 (4)	0.012 (4)	0.039 (4)	0.005 (3)
C16	0.074 (3)	0.058 (2)	0.069 (3)	0.0075 (18)	0.020 (2)	0.0015 (18)
C17	0.095 (4)	0.080 (3)	0.061 (3)	−0.003 (2)	0.025 (2)	−0.007 (2)
C18	0.083 (3)	0.098 (4)	0.067 (3)	0.004 (3)	0.004 (2)	0.004 (2)
C19	0.071 (3)	0.086 (3)	0.084 (3)	0.003 (2)	0.003 (2)	0.005 (2)
C20	0.071 (3)	0.065 (2)	0.082 (3)	0.0051 (19)	0.021 (2)	0.002 (2)
C21	0.066 (3)	0.066 (2)	0.065 (3)	−0.0011 (18)	0.0158 (19)	−0.0019 (18)
C22	0.081 (4)	0.096 (4)	0.116 (5)	0.015 (3)	0.038 (3)	0.015 (4)

### 2,3-Bis[3-(trifluoromethyl)phenyl]-2,3-dihydro-4H-1,3-benzothiazin-4-one (1) Geometric parameters (Å, º)

**Table:**

S1—C1	1.784 (4)	C6—C7	1.377 (10)
S1—C4	1.736 (6)	C7—H7	0.9300
F1—C15	1.317 (11)	C7—C8	1.376 (9)
F1A—C15	1.35 (2)	C8—H8	0.9300
F2—C15	1.393 (12)	C9—C10	1.375 (6)
F2A—C15	1.20 (3)	C9—C14	1.400 (6)
F3—C15	1.285 (10)	C10—H10	0.9300
F3A—C15	1.151 (14)	C10—C11	1.384 (6)
F4—C22	1.343 (14)	C11—C12	1.388 (6)
F4A—C22	1.14 (3)	C11—C15	1.493 (7)
F5—C22	1.408 (9)	C12—H12	0.9300
F5A—C22	1.238 (17)	C12—C13	1.363 (7)
F6—C22	1.258 (10)	C13—H13	0.9300
F6A—C22	1.428 (15)	C13—C14	1.376 (7)
O1—C2	1.210 (5)	C14—H14	0.9300
N1—C1	1.478 (5)	C16—C17	1.387 (6)
N1—C2	1.385 (5)	C16—C21	1.397 (6)
N1—C16	1.420 (5)	C17—H17	0.9300
C1—H1	0.9800	C17—C18	1.364 (6)
C1—C9	1.483 (6)	C18—H18	0.9300
C2—C3	1.481 (6)	C18—C19	1.365 (7)
C3—C4	1.401 (7)	C19—H19	0.9300
C3—C8	1.398 (7)	C19—C20	1.380 (6)
C4—C5	1.408 (8)	C20—C21	1.378 (6)
C5—H5	0.9300	C20—C22	1.473 (7)
C5—C6	1.362 (10)	C21—H21	0.9300
C6—H6	0.9300		
			
C4—S1—C1	95.2 (2)	F2A—C15—F1	46 (2)
C2—N1—C1	119.6 (3)	F2A—C15—F1A	104 (2)
C2—N1—C16	119.8 (3)	F2A—C15—F2	56 (2)
C16—N1—C1	119.8 (3)	F2A—C15—F3	130.6 (15)
S1—C1—H1	107.7	F2A—C15—C11	112.4 (13)
N1—C1—S1	110.7 (3)	F3—C15—F1	108.0 (9)
N1—C1—H1	107.7	F3—C15—F1A	60.0 (16)
N1—C1—C9	112.8 (3)	F3—C15—F2	106.2 (10)
C9—C1—S1	109.9 (3)	F3—C15—C11	116.9 (8)
C9—C1—H1	107.7	F3A—C15—F1	127.8 (12)
O1—C2—N1	120.8 (4)	F3A—C15—F1A	108.4 (19)
O1—C2—C3	122.1 (4)	F3A—C15—F2	58.2 (17)
N1—C2—C3	116.7 (4)	F3A—C15—F2A	107 (2)
C4—C3—C2	123.6 (4)	F3A—C15—F3	50.8 (16)
C8—C3—C2	117.9 (5)	F3A—C15—C11	116.7 (12)
C8—C3—C4	118.3 (5)	C17—C16—N1	121.0 (4)
C3—C4—S1	122.3 (4)	C17—C16—C21	119.3 (4)
C3—C4—C5	118.9 (5)	C21—C16—N1	119.6 (4)
C5—C4—S1	118.6 (5)	C16—C17—H17	119.8
C4—C5—H5	119.4	C18—C17—C16	120.4 (4)
C6—C5—C4	121.1 (7)	C18—C17—H17	119.8
C6—C5—H5	119.4	C17—C18—H18	119.8
C5—C6—H6	119.9	C17—C18—C19	120.4 (4)
C5—C6—C7	120.2 (7)	C19—C18—H18	119.8
C7—C6—H6	119.9	C18—C19—H19	119.9
C6—C7—H7	120.1	C18—C19—C20	120.3 (4)
C8—C7—C6	119.9 (7)	C20—C19—H19	119.9
C8—C7—H7	120.1	C19—C20—C22	119.7 (5)
C3—C8—H8	119.3	C21—C20—C19	120.2 (4)
C7—C8—C3	121.4 (7)	C21—C20—C22	120.1 (5)
C7—C8—H8	119.3	C16—C21—H21	120.4
C10—C9—C1	119.1 (4)	C20—C21—C16	119.3 (4)
C10—C9—C14	117.8 (4)	C20—C21—H21	120.4
C14—C9—C1	123.1 (4)	F4—C22—F5	98.0 (8)
C9—C10—H10	119.8	F4—C22—F6A	72.6 (10)
C9—C10—C11	120.4 (4)	F4—C22—C20	114.3 (6)
C11—C10—H10	119.8	F4A—C22—F4	32 (2)
C10—C11—C12	121.0 (4)	F4A—C22—F5	67 (2)
C10—C11—C15	120.6 (4)	F4A—C22—F5A	111 (2)
C12—C11—C15	118.4 (4)	F4A—C22—F6	128.8 (19)
C11—C12—H12	120.5	F4A—C22—F6A	103 (2)
C13—C12—C11	119.0 (4)	F4A—C22—C20	115.7 (17)
C13—C12—H12	120.5	F5—C22—F6A	143.9 (7)
C12—C13—H13	119.9	F5—C22—C20	108.4 (6)
C12—C13—C14	120.2 (4)	F5A—C22—F4	126.9 (12)
C14—C13—H13	119.9	F5A—C22—F5	58.2 (14)
C9—C14—H14	119.2	F5A—C22—F6	48.3 (11)
C13—C14—C9	121.5 (4)	F5A—C22—F6A	99.0 (13)
C13—C14—H14	119.2	F5A—C22—C20	118.1 (11)
F1—C15—F1A	59.2 (19)	F6—C22—F4	114.2 (11)
F1—C15—F2	99.0 (10)	F6—C22—F5	105.3 (10)
F1—C15—C11	115.1 (6)	F6—C22—F6A	52.8 (8)
F1A—C15—F2	142.1 (11)	F6—C22—C20	114.6 (7)
F1A—C15—C11	107.7 (10)	F6A—C22—C20	107.1 (7)
F2—C15—C11	109.8 (6)		
			
S1—C1—C9—C10	−131.6 (4)	C10—C11—C15—F1A	−89 (3)
S1—C1—C9—C14	49.1 (5)	C10—C11—C15—F2	85.3 (13)
S1—C4—C5—C6	−178.4 (6)	C10—C11—C15—F2A	25 (3)
O1—C2—C3—C4	157.0 (5)	C10—C11—C15—F3	−153.7 (14)
O1—C2—C3—C8	−17.7 (6)	C10—C11—C15—F3A	149 (3)
N1—C1—C9—C10	104.3 (4)	C11—C12—C13—C14	2.6 (7)
N1—C1—C9—C14	−75.1 (5)	C12—C11—C15—F1	153.3 (10)
N1—C2—C3—C4	−16.9 (6)	C12—C11—C15—F1A	90 (3)
N1—C2—C3—C8	168.4 (4)	C12—C11—C15—F2	−96.1 (13)
N1—C16—C17—C18	−175.7 (4)	C12—C11—C15—F2A	−157 (3)
N1—C16—C21—C20	176.0 (4)	C12—C11—C15—F3	24.9 (15)
C1—S1—C4—C3	29.4 (5)	C12—C11—C15—F3A	−33 (3)
C1—S1—C4—C5	−156.4 (5)	C12—C13—C14—C9	−2.1 (7)
C1—N1—C2—O1	168.2 (4)	C14—C9—C10—C11	0.7 (6)
C1—N1—C2—C3	−17.8 (5)	C15—C11—C12—C13	180.0 (5)
C1—N1—C16—C17	105.6 (4)	C16—N1—C1—S1	−131.0 (3)
C1—N1—C16—C21	−72.5 (5)	C16—N1—C1—C9	−7.3 (5)
C1—C9—C10—C11	−178.7 (4)	C16—N1—C2—O1	−1.6 (6)
C1—C9—C14—C13	179.8 (4)	C16—N1—C2—C3	172.4 (4)
C2—N1—C1—S1	59.2 (4)	C16—C17—C18—C19	−1.0 (7)
C2—N1—C1—C9	−177.1 (3)	C17—C16—C21—C20	−2.1 (6)
C2—N1—C16—C17	−84.7 (5)	C17—C18—C19—C20	−0.8 (7)
C2—N1—C16—C21	97.2 (5)	C18—C19—C20—C21	1.1 (7)
C2—C3—C4—S1	5.1 (7)	C18—C19—C20—C22	179.2 (5)
C2—C3—C4—C5	−169.1 (5)	C19—C20—C21—C16	0.4 (6)
C2—C3—C8—C7	171.5 (5)	C19—C20—C22—F4	173.1 (8)
C3—C4—C5—C6	−4.0 (10)	C19—C20—C22—F4A	137 (3)
C4—S1—C1—N1	−57.7 (3)	C19—C20—C22—F5	65.0 (9)
C4—S1—C1—C9	177.0 (3)	C19—C20—C22—F5A	2 (2)
C4—C3—C8—C7	−3.5 (8)	C19—C20—C22—F6	−52.4 (14)
C4—C5—C6—C7	0.1 (12)	C19—C20—C22—F6A	−108.7 (11)
C5—C6—C7—C8	2.1 (12)	C21—C16—C17—C18	2.4 (6)
C6—C7—C8—C3	−0.4 (10)	C21—C20—C22—F4	−8.8 (10)
C8—C3—C4—S1	179.8 (4)	C21—C20—C22—F4A	−45 (3)
C8—C3—C4—C5	5.6 (8)	C21—C20—C22—F5	−116.9 (8)
C9—C10—C11—C12	−0.2 (7)	C21—C20—C22—F5A	180 (2)
C9—C10—C11—C15	178.3 (5)	C21—C20—C22—F6	125.8 (13)
C10—C9—C14—C13	0.4 (7)	C21—C20—C22—F6A	69.5 (11)
C10—C11—C12—C13	−1.5 (7)	C22—C20—C21—C16	−177.7 (4)
C10—C11—C15—F1	−25.3 (12)		

### 2,3-Bis[3-(trifluoromethyl)phenyl]-2,3-dihydro-4H-1,3-benzothiazin-4-one (1) Hydrogen-bond geometry (Å, º)

**Table:**

*D*—H···*A*	*D*—H	H···*A*	*D*···*A*	*D*—H···*A*
C1—H1···O1^i^	0.98	2.61	3.360 (5)	133
C10—H10···O1^i^	0.93	2.59	3.370 (5)	141
C14—H14···F2*A*^ii^	0.93	2.59	3.27 (2)	130
C18—H18···F4^ii^	0.93	2.56	3.394 (10)	149
C21—H21···O1^i^	0.93	2.50	3.368 (6)	156

Symmetry codes: (i) *x*, −*y*+3/2, *z*+1/2; (ii) *x*, *y*, *z*−1.

### 2,3-Bis(4-methylphenyl)-2,3-dihydro-4H-1,3-benzothiazin-4-one (2) Crystal data

**Table:**

C_22_H_19_NOS	*F*(000) = 2912
*M**_r_* = 345.44	*D*_x_ = 1.256 Mg m^−^^3^
Monoclinic, *C*2/*c*	Mo *K*α radiation, λ = 0.71073 Å
*a* = 24.821 (7) Å	Cell parameters from 6387 reflections
*b* = 12.151 (3) Å	θ = 2.2–26.5°
*c* = 26.219 (7) Å	µ = 0.19 mm^−^^1^
β = 112.470 (4)°	*T* = 298 K
*V* = 7307 (3) Å^3^	Block, colorless
*Z* = 16	0.29 × 0.28 × 0.2 mm

### 2,3-Bis(4-methylphenyl)-2,3-dihydro-4H-1,3-benzothiazin-4-one (2) Data collection

**Table:**

Bruker SMART CCD diffractometer	8985 independent reflections
Radiation source: fine-focus sealed tube	5453 reflections with *I* > 2σ(*I*)
Graphite monochromator	*R*_int_ = 0.046
Detector resolution: 8.34 pixels mm^-1^	θ_max_ = 28.3°, θ_min_ = 1.7°
phi and ω scans	*h* = −32→30
Absorption correction: multi-scan (SADABS; Bruker, 2001)	*k* = −16→11
*T*_min_ = 0.100, *T*_max_ = 0.85	*l* = −34→34
26265 measured reflections	

### 2,3-Bis(4-methylphenyl)-2,3-dihydro-4H-1,3-benzothiazin-4-one (2) Refinement

**Table:**

Refinement on *F*^2^	Primary atom site location: structure-invariant direct methods
Least-squares matrix: full	Secondary atom site location: difference Fourier map
*R*[*F*^2^ > 2σ(*F*^2^)] = 0.058	Hydrogen site location: inferred from neighbouring sites
*wR*(*F*^2^) = 0.174	H-atom parameters constrained
*S* = 0.96	*w* = 1/[σ^2^(*F*_o_^2^) + (0.1*P*)^2^] where *P* = (*F*_o_^2^ + 2*F*_c_^2^)/3
8985 reflections	(Δ/σ)_max_ < 0.001
455 parameters	Δρ_max_ = 0.32 e Å^−^^3^
0 restraints	Δρ_min_ = −0.33 e Å^−^^3^

### 2,3-Bis(4-methylphenyl)-2,3-dihydro-4H-1,3-benzothiazin-4-one (2) Special details

**Table:**

Experimental. The data collection nominally covered a full sphere of reciprocal space by a combination of 4 sets of ω scans each set at different φ and/or 2θ angles and each scan (10 s exposure) covering -0.300° degrees in ω. The crystal to detector distance was 5.82 cm.
Geometry. All esds (except the esd in the dihedral angle between two l.s. planes) are estimated using the full covariance matrix. The cell esds are taken into account individually in the estimation of esds in distances, angles and torsion angles; correlations between esds in cell parameters are only used when they are defined by crystal symmetry. An approximate (isotropic) treatment of cell esds is used for estimating esds involving l.s. planes.
Refinement. Refinement of F^2^ against ALL reflections. The weighted R-factor wR and goodness of fit S are based on F^2^, conventional R-factors R are based on F, with F set to zero for negative F^2^. The threshold expression of F^2^ > 2sigma(F^2^) is used only for calculating R-factors(gt) etc. and is not relevant to the choice of reflections for refinement. R-factors based on F^2^ are statistically about twice as large as those based on F, and R- factors based on ALL data will be even larger.

### 2,3-Bis(4-methylphenyl)-2,3-dihydro-4H-1,3-benzothiazin-4-one (2) Fractional atomic coordinates and isotropic or equivalent isotropic displacement parameters (Å^2^)

**Table:**

	*x*	*y*	*z*	*U*_iso_*/*U*_eq_	
C1	0.69203 (9)	0.43913 (16)	0.38181 (8)	0.0537 (5)	
H1	0.6853	0.3881	0.4077	0.064*	
C2	0.71007 (9)	0.64058 (16)	0.38441 (9)	0.0554 (5)	
C3	0.74576 (9)	0.62403 (17)	0.35099 (9)	0.0583 (5)	
C4	0.77404 (9)	0.52539 (19)	0.34957 (9)	0.0620 (6)	
C5	0.81323 (11)	0.5215 (3)	0.32349 (12)	0.0829 (8)	
H5	0.8327	0.4562	0.3234	0.099*	
C6	0.82346 (14)	0.6118 (3)	0.29810 (13)	0.0991 (10)	
H6	0.8499	0.6078	0.2808	0.119*	
C7	0.79488 (14)	0.7100 (3)	0.29771 (12)	0.0967 (9)	
H7	0.8015	0.7714	0.2797	0.116*	
C8	0.75636 (12)	0.7158 (2)	0.32431 (10)	0.0754 (7)	
H8	0.7373	0.7817	0.3244	0.090*	
C9	0.64495 (8)	0.41536 (15)	0.32575 (8)	0.0505 (5)	
C10	0.63218 (10)	0.30700 (17)	0.31082 (9)	0.0625 (6)	
H10	0.6523	0.2517	0.3352	0.075*	
C11	0.59011 (10)	0.2790 (2)	0.26034 (10)	0.0718 (6)	
H11	0.5819	0.2050	0.2519	0.086*	
C12	0.56009 (9)	0.3567 (2)	0.22225 (10)	0.0695 (6)	
C13	0.57233 (12)	0.4638 (2)	0.23774 (12)	0.0974 (10)	
H13	0.5523	0.5187	0.2131	0.117*	
C14	0.61355 (11)	0.4943 (2)	0.28892 (12)	0.0887 (9)	
H14	0.6198	0.5684	0.2982	0.106*	
C15	0.51477 (12)	0.3251 (3)	0.16630 (11)	0.1052 (10)	
H15A	0.5279	0.2617	0.1525	0.158*	
H15B	0.5091	0.3853	0.1411	0.158*	
H15C	0.4786	0.3085	0.1699	0.158*	
C16	0.66457 (9)	0.56341 (15)	0.44320 (9)	0.0524 (5)	
C17	0.69408 (9)	0.61834 (16)	0.49170 (9)	0.0568 (5)	
H17	0.7309	0.6469	0.4985	0.068*	
C18	0.66956 (10)	0.63141 (18)	0.53031 (9)	0.0622 (6)	
H18	0.6902	0.6684	0.5630	0.075*	
C19	0.61458 (11)	0.59039 (19)	0.52124 (11)	0.0691 (6)	
C20	0.58631 (11)	0.5337 (2)	0.47281 (12)	0.0772 (7)	
H20	0.5496	0.5045	0.4661	0.093*	
C21	0.61053 (10)	0.51887 (19)	0.43411 (11)	0.0699 (6)	
H21	0.5907	0.4792	0.4021	0.084*	
C22	0.58743 (14)	0.6089 (3)	0.56288 (13)	0.1016 (10)	
H22A	0.6164	0.5989	0.5994	0.152*	
H22B	0.5563	0.5572	0.5566	0.152*	
H22C	0.5723	0.6825	0.5592	0.152*	
N1	0.69068 (7)	0.54944 (13)	0.40328 (7)	0.0539 (4)	
O1	0.69975 (7)	0.73376 (11)	0.39598 (7)	0.0718 (4)	
S1	0.76424 (2)	0.40690 (5)	0.38303 (3)	0.0683 (2)	
C23	0.63847 (8)	−0.06355 (14)	0.43575 (7)	0.0456 (4)	
H23	0.6665	−0.1129	0.4294	0.055*	
C24	0.63691 (9)	0.13713 (15)	0.45273 (8)	0.0496 (5)	
C25	0.60023 (8)	0.11523 (15)	0.48487 (8)	0.0483 (4)	
C26	0.60103 (8)	0.01584 (16)	0.51208 (7)	0.0489 (5)	
C27	0.57311 (10)	0.0090 (2)	0.54882 (8)	0.0626 (6)	
H27	0.5749	−0.0562	0.5680	0.075*	
C28	0.54295 (10)	0.0972 (2)	0.55722 (10)	0.0737 (7)	
H28	0.5245	0.0915	0.5820	0.088*	
C29	0.53988 (10)	0.1944 (2)	0.52887 (10)	0.0754 (7)	
H29	0.5185	0.2534	0.5338	0.091*	
C30	0.56844 (9)	0.20366 (19)	0.49332 (9)	0.0633 (6)	
H30	0.5666	0.2695	0.4746	0.076*	
C31	0.57976 (8)	−0.08712 (14)	0.39016 (7)	0.0439 (4)	
C32	0.56036 (9)	−0.19472 (16)	0.38227 (8)	0.0548 (5)	
H32	0.5833	−0.2498	0.4048	0.066*	
C33	0.50727 (9)	−0.22179 (19)	0.34125 (9)	0.0617 (5)	
H33	0.4952	−0.2948	0.3370	0.074*	
C34	0.47204 (9)	−0.14356 (19)	0.30666 (8)	0.0563 (5)	
C35	0.49174 (9)	−0.03677 (19)	0.31450 (8)	0.0615 (5)	
H35	0.4686	0.0179	0.2917	0.074*	
C36	0.54490 (9)	−0.00759 (16)	0.35518 (8)	0.0545 (5)	
H36	0.5571	0.0654	0.3589	0.065*	
C37	0.41430 (10)	−0.1747 (2)	0.26196 (9)	0.0809 (8)	
H37A	0.4210	−0.2060	0.2313	0.121*	
H37B	0.3904	−0.1102	0.2500	0.121*	
H37C	0.3949	−0.2275	0.2762	0.121*	
C38	0.70360 (8)	0.06513 (15)	0.41266 (8)	0.0473 (4)	
C39	0.69724 (9)	0.01669 (17)	0.36311 (8)	0.0576 (5)	
H39	0.6649	−0.0271	0.3444	0.069*	
C40	0.73937 (11)	0.0338 (2)	0.34142 (10)	0.0724 (7)	
H40	0.7351	0.0005	0.3082	0.087*	
C41	0.78753 (11)	0.0989 (2)	0.36779 (12)	0.0747 (7)	
C42	0.79376 (10)	0.14326 (19)	0.41859 (11)	0.0704 (6)	
H42	0.8267	0.1850	0.4380	0.084*	
C43	0.75286 (9)	0.12709 (16)	0.44072 (9)	0.0573 (5)	
H43	0.7581	0.1578	0.4748	0.069*	
C44	0.83113 (14)	0.1224 (3)	0.34151 (15)	0.1201 (12)	
H44A	0.8307	0.0632	0.3171	0.180*	
H44B	0.8694	0.1292	0.3698	0.180*	
H44C	0.8208	0.1899	0.3210	0.180*	
N2	0.66062 (7)	0.04828 (12)	0.43611 (6)	0.0465 (4)	
O2	0.64754 (7)	0.23142 (11)	0.44332 (6)	0.0678 (4)	
S2	0.63943 (2)	−0.09836 (4)	0.50364 (2)	0.05417 (17)	

### 2,3-Bis(4-methylphenyl)-2,3-dihydro-4H-1,3-benzothiazin-4-one (2) Atomic displacement parameters (Å^2^)

**Table:**

	*U*^11^	*U*^22^	*U*^33^	*U*^12^	*U*^13^	*U*^23^
C1	0.0599 (12)	0.0329 (10)	0.0653 (12)	0.0020 (9)	0.0204 (10)	−0.0012 (8)
C2	0.0560 (12)	0.0385 (11)	0.0750 (14)	−0.0016 (9)	0.0286 (11)	−0.0044 (10)
C3	0.0598 (12)	0.0487 (12)	0.0675 (13)	−0.0064 (10)	0.0257 (11)	−0.0112 (10)
C4	0.0472 (11)	0.0687 (15)	0.0640 (13)	−0.0005 (10)	0.0145 (10)	−0.0168 (11)
C5	0.0703 (16)	0.093 (2)	0.0903 (19)	0.0011 (14)	0.0363 (14)	−0.0239 (16)
C6	0.097 (2)	0.121 (3)	0.098 (2)	−0.016 (2)	0.0573 (19)	−0.025 (2)
C7	0.112 (2)	0.102 (2)	0.093 (2)	−0.028 (2)	0.0587 (18)	−0.0087 (17)
C8	0.0900 (17)	0.0667 (16)	0.0801 (16)	−0.0135 (13)	0.0443 (14)	−0.0061 (12)
C9	0.0505 (11)	0.0419 (11)	0.0613 (12)	0.0008 (8)	0.0240 (9)	0.0054 (9)
C10	0.0744 (14)	0.0440 (12)	0.0622 (13)	0.0062 (10)	0.0184 (11)	−0.0029 (9)
C11	0.0758 (15)	0.0628 (15)	0.0705 (15)	0.0017 (12)	0.0210 (12)	−0.0155 (12)
C12	0.0528 (13)	0.0935 (19)	0.0638 (14)	−0.0045 (13)	0.0242 (11)	0.0013 (13)
C13	0.0771 (17)	0.086 (2)	0.097 (2)	−0.0040 (15)	−0.0033 (15)	0.0405 (16)
C14	0.0775 (16)	0.0513 (14)	0.103 (2)	−0.0058 (12)	−0.0039 (15)	0.0209 (13)
C15	0.0727 (17)	0.162 (3)	0.0672 (16)	−0.0157 (19)	0.0113 (14)	−0.0036 (18)
C16	0.0529 (11)	0.0340 (10)	0.0706 (13)	0.0001 (9)	0.0240 (10)	0.0026 (9)
C17	0.0518 (11)	0.0449 (11)	0.0717 (14)	0.0029 (9)	0.0214 (11)	0.0034 (10)
C18	0.0692 (14)	0.0507 (13)	0.0652 (13)	0.0101 (11)	0.0241 (11)	0.0056 (10)
C19	0.0734 (15)	0.0563 (14)	0.0862 (17)	0.0118 (12)	0.0401 (14)	0.0145 (12)
C20	0.0647 (14)	0.0688 (16)	0.107 (2)	−0.0104 (13)	0.0428 (15)	0.0027 (15)
C21	0.0627 (14)	0.0587 (14)	0.0902 (17)	−0.0162 (11)	0.0314 (13)	−0.0136 (12)
C22	0.110 (2)	0.113 (3)	0.105 (2)	0.0228 (19)	0.069 (2)	0.0217 (18)
N1	0.0616 (10)	0.0333 (9)	0.0711 (11)	−0.0028 (7)	0.0302 (9)	−0.0030 (7)
O1	0.0907 (11)	0.0344 (8)	0.1102 (13)	−0.0027 (7)	0.0605 (10)	−0.0048 (8)
S1	0.0560 (3)	0.0515 (3)	0.0850 (4)	0.0120 (3)	0.0131 (3)	−0.0085 (3)
C23	0.0511 (10)	0.0333 (9)	0.0518 (10)	0.0012 (8)	0.0190 (9)	−0.0019 (8)
C24	0.0622 (12)	0.0357 (10)	0.0507 (10)	−0.0008 (9)	0.0215 (9)	−0.0013 (8)
C25	0.0505 (11)	0.0446 (11)	0.0466 (10)	0.0015 (8)	0.0151 (9)	−0.0037 (8)
C26	0.0482 (10)	0.0543 (12)	0.0396 (9)	−0.0021 (9)	0.0116 (8)	−0.0038 (8)
C27	0.0664 (13)	0.0720 (15)	0.0490 (11)	−0.0074 (11)	0.0218 (10)	0.0003 (10)
C28	0.0637 (14)	0.103 (2)	0.0597 (14)	−0.0045 (14)	0.0289 (12)	−0.0125 (13)
C29	0.0691 (15)	0.0851 (19)	0.0761 (16)	0.0135 (13)	0.0324 (13)	−0.0165 (14)
C30	0.0673 (13)	0.0574 (13)	0.0652 (13)	0.0121 (11)	0.0255 (11)	−0.0033 (10)
C31	0.0515 (10)	0.0358 (10)	0.0474 (10)	0.0014 (8)	0.0222 (8)	−0.0012 (7)
C32	0.0569 (12)	0.0394 (11)	0.0631 (12)	0.0019 (9)	0.0172 (10)	−0.0016 (9)
C33	0.0611 (12)	0.0526 (12)	0.0692 (13)	−0.0099 (10)	0.0224 (11)	−0.0129 (10)
C34	0.0525 (11)	0.0703 (15)	0.0479 (11)	−0.0006 (10)	0.0213 (9)	−0.0054 (10)
C35	0.0602 (13)	0.0687 (15)	0.0500 (11)	0.0088 (11)	0.0148 (10)	0.0086 (10)
C36	0.0628 (13)	0.0448 (11)	0.0533 (11)	0.0006 (9)	0.0193 (10)	0.0042 (9)
C37	0.0628 (14)	0.109 (2)	0.0610 (14)	−0.0087 (14)	0.0124 (11)	−0.0117 (13)
C38	0.0509 (11)	0.0389 (10)	0.0518 (10)	0.0011 (8)	0.0192 (9)	0.0018 (8)
C39	0.0607 (12)	0.0590 (13)	0.0560 (12)	−0.0046 (10)	0.0253 (10)	−0.0039 (10)
C40	0.0881 (17)	0.0731 (16)	0.0668 (14)	0.0089 (14)	0.0415 (14)	0.0102 (12)
C41	0.0670 (15)	0.0662 (16)	0.103 (2)	0.0093 (12)	0.0463 (15)	0.0281 (14)
C42	0.0585 (13)	0.0553 (14)	0.0948 (18)	−0.0043 (11)	0.0266 (13)	0.0115 (13)
C43	0.0567 (12)	0.0439 (11)	0.0657 (13)	−0.0038 (9)	0.0172 (10)	0.0000 (9)
C44	0.107 (2)	0.126 (3)	0.164 (3)	0.013 (2)	0.092 (3)	0.049 (2)
N2	0.0555 (9)	0.0333 (8)	0.0537 (9)	−0.0032 (7)	0.0244 (8)	−0.0036 (7)
O2	0.0971 (11)	0.0352 (8)	0.0838 (10)	−0.0006 (8)	0.0485 (9)	0.0009 (7)
S2	0.0635 (3)	0.0438 (3)	0.0498 (3)	0.0034 (2)	0.0157 (2)	0.0073 (2)

### 2,3-Bis(4-methylphenyl)-2,3-dihydro-4H-1,3-benzothiazin-4-one (2) Geometric parameters (Å, º)

**Table:**

C1—H1	0.9800	C23—H23	0.9800
C1—C9	1.515 (3)	C23—C31	1.518 (2)
C1—N1	1.459 (2)	C23—N2	1.465 (2)
C1—S1	1.823 (2)	C23—S2	1.821 (2)
C2—C3	1.479 (3)	C24—C25	1.482 (3)
C2—N1	1.373 (3)	C24—N2	1.378 (2)
C2—O1	1.224 (2)	C24—O2	1.222 (2)
C3—C4	1.397 (3)	C25—C26	1.399 (3)
C3—C8	1.394 (3)	C25—C30	1.400 (3)
C4—C5	1.387 (3)	C26—C27	1.388 (3)
C4—S1	1.750 (3)	C26—S2	1.745 (2)
C5—H5	0.9300	C27—H27	0.9300
C5—C6	1.357 (4)	C27—C28	1.372 (3)
C6—H6	0.9300	C28—H28	0.9300
C6—C7	1.386 (4)	C28—C29	1.382 (3)
C7—H7	0.9300	C29—H29	0.9300
C7—C8	1.384 (4)	C29—C30	1.374 (3)
C8—H8	0.9300	C30—H30	0.9300
C9—C10	1.376 (3)	C31—C32	1.381 (3)
C9—C14	1.373 (3)	C31—C36	1.385 (3)
C10—H10	0.9300	C32—H32	0.9300
C10—C11	1.379 (3)	C32—C33	1.385 (3)
C11—H11	0.9300	C33—H33	0.9300
C11—C12	1.369 (3)	C33—C34	1.373 (3)
C12—C13	1.363 (4)	C34—C35	1.374 (3)
C12—C15	1.517 (3)	C34—C37	1.512 (3)
C13—H13	0.9300	C35—H35	0.9300
C13—C14	1.391 (4)	C35—C36	1.389 (3)
C14—H14	0.9300	C36—H36	0.9300
C15—H15A	0.9600	C37—H37A	0.9600
C15—H15B	0.9600	C37—H37B	0.9600
C15—H15C	0.9600	C37—H37C	0.9600
C16—C17	1.374 (3)	C38—C39	1.379 (3)
C16—C21	1.380 (3)	C38—C43	1.384 (3)
C16—N1	1.437 (3)	C38—N2	1.435 (2)
C17—H17	0.9300	C39—H39	0.9300
C17—C18	1.375 (3)	C39—C40	1.384 (3)
C18—H18	0.9300	C40—H40	0.9300
C18—C19	1.385 (3)	C40—C41	1.378 (4)
C19—C20	1.378 (4)	C41—C42	1.390 (4)
C19—C22	1.503 (4)	C41—C44	1.516 (4)
C20—H20	0.9300	C42—H42	0.9300
C20—C21	1.375 (3)	C42—C43	1.362 (3)
C21—H21	0.9300	C43—H43	0.9300
C22—H22A	0.9600	C44—H44A	0.9600
C22—H22B	0.9600	C44—H44B	0.9600
C22—H22C	0.9600	C44—H44C	0.9600
			
C9—C1—H1	106.2	C31—C23—H23	106.3
C9—C1—S1	111.38 (14)	C31—C23—S2	112.08 (13)
N1—C1—H1	106.2	N2—C23—H23	106.3
N1—C1—C9	115.24 (16)	N2—C23—C31	115.16 (15)
N1—C1—S1	110.97 (13)	N2—C23—S2	110.17 (12)
S1—C1—H1	106.2	S2—C23—H23	106.3
N1—C2—C3	118.41 (18)	N2—C24—C25	117.95 (16)
O1—C2—C3	120.15 (19)	O2—C24—C25	120.68 (18)
O1—C2—N1	121.41 (19)	O2—C24—N2	121.31 (18)
C4—C3—C2	123.6 (2)	C26—C25—C24	123.57 (17)
C8—C3—C2	117.3 (2)	C26—C25—C30	118.74 (19)
C8—C3—C4	118.7 (2)	C30—C25—C24	117.26 (18)
C3—C4—S1	121.68 (18)	C25—C26—S2	121.60 (15)
C5—C4—C3	119.8 (2)	C27—C26—C25	119.51 (19)
C5—C4—S1	118.5 (2)	C27—C26—S2	118.82 (16)
C4—C5—H5	119.6	C26—C27—H27	119.6
C6—C5—C4	120.8 (3)	C28—C27—C26	120.8 (2)
C6—C5—H5	119.6	C28—C27—H27	119.6
C5—C6—H6	119.7	C27—C28—H28	119.9
C5—C6—C7	120.6 (3)	C27—C28—C29	120.2 (2)
C7—C6—H6	119.7	C29—C28—H28	119.9
C6—C7—H7	120.3	C28—C29—H29	120.1
C8—C7—C6	119.3 (3)	C30—C29—C28	119.9 (2)
C8—C7—H7	120.3	C30—C29—H29	120.1
C3—C8—H8	119.6	C25—C30—H30	119.6
C7—C8—C3	120.8 (3)	C29—C30—C25	120.8 (2)
C7—C8—H8	119.6	C29—C30—H30	119.6
C10—C9—C1	117.82 (17)	C32—C31—C23	118.12 (16)
C14—C9—C1	124.68 (19)	C32—C31—C36	117.88 (18)
C14—C9—C10	117.5 (2)	C36—C31—C23	123.98 (16)
C9—C10—H10	119.5	C31—C32—H32	119.5
C9—C10—C11	121.1 (2)	C31—C32—C33	120.98 (19)
C11—C10—H10	119.5	C33—C32—H32	119.5
C10—C11—H11	118.9	C32—C33—H33	119.1
C12—C11—C10	122.1 (2)	C34—C33—C32	121.7 (2)
C12—C11—H11	118.9	C34—C33—H33	119.1
C11—C12—C15	121.8 (3)	C33—C34—C35	117.03 (19)
C13—C12—C11	116.3 (2)	C33—C34—C37	121.0 (2)
C13—C12—C15	121.8 (3)	C35—C34—C37	122.0 (2)
C12—C13—H13	118.7	C34—C35—H35	118.8
C12—C13—C14	122.7 (2)	C34—C35—C36	122.4 (2)
C14—C13—H13	118.7	C36—C35—H35	118.8
C9—C14—C13	120.2 (2)	C31—C36—C35	120.02 (19)
C9—C14—H14	119.9	C31—C36—H36	120.0
C13—C14—H14	119.9	C35—C36—H36	120.0
C12—C15—H15A	109.5	C34—C37—H37A	109.5
C12—C15—H15B	109.5	C34—C37—H37B	109.5
C12—C15—H15C	109.5	C34—C37—H37C	109.5
H15A—C15—H15B	109.5	H37A—C37—H37B	109.5
H15A—C15—H15C	109.5	H37A—C37—H37C	109.5
H15B—C15—H15C	109.5	H37B—C37—H37C	109.5
C17—C16—C21	119.3 (2)	C39—C38—C43	119.51 (19)
C17—C16—N1	120.17 (18)	C39—C38—N2	120.43 (17)
C21—C16—N1	120.5 (2)	C43—C38—N2	120.02 (18)
C16—C17—H17	119.7	C38—C39—H39	120.3
C16—C17—C18	120.5 (2)	C38—C39—C40	119.4 (2)
C18—C17—H17	119.7	C40—C39—H39	120.3
C17—C18—H18	119.5	C39—C40—H40	119.1
C17—C18—C19	121.1 (2)	C41—C40—C39	121.8 (2)
C19—C18—H18	119.5	C41—C40—H40	119.1
C18—C19—C22	120.5 (3)	C40—C41—C42	117.4 (2)
C20—C19—C18	117.4 (2)	C40—C41—C44	120.9 (3)
C20—C19—C22	122.2 (3)	C42—C41—C44	121.7 (3)
C19—C20—H20	118.9	C41—C42—H42	119.2
C21—C20—C19	122.2 (2)	C43—C42—C41	121.6 (2)
C21—C20—H20	118.9	C43—C42—H42	119.2
C16—C21—H21	120.3	C38—C43—H43	119.9
C20—C21—C16	119.4 (2)	C42—C43—C38	120.2 (2)
C20—C21—H21	120.3	C42—C43—H43	119.9
C19—C22—H22A	109.5	C41—C44—H44A	109.5
C19—C22—H22B	109.5	C41—C44—H44B	109.5
C19—C22—H22C	109.5	C41—C44—H44C	109.5
H22A—C22—H22B	109.5	H44A—C44—H44B	109.5
H22A—C22—H22C	109.5	H44A—C44—H44C	109.5
H22B—C22—H22C	109.5	H44B—C44—H44C	109.5
C2—N1—C1	122.63 (17)	C24—N2—C23	121.63 (16)
C2—N1—C16	119.03 (16)	C24—N2—C38	120.08 (15)
C16—N1—C1	118.23 (16)	C38—N2—C23	117.93 (14)
C4—S1—C1	97.96 (10)	C26—S2—C23	97.79 (9)
			
C1—C9—C10—C11	−179.6 (2)	C23—C31—C32—C33	−179.76 (18)
C1—C9—C14—C13	178.1 (2)	C23—C31—C36—C35	179.92 (18)
C2—C3—C4—C5	−170.8 (2)	C24—C25—C26—C27	168.90 (18)
C2—C3—C4—S1	5.9 (3)	C24—C25—C26—S2	−7.9 (3)
C2—C3—C8—C7	172.3 (2)	C24—C25—C30—C29	−170.92 (19)
C3—C2—N1—C1	−14.5 (3)	C25—C24—N2—C23	16.9 (2)
C3—C2—N1—C16	169.61 (18)	C25—C24—N2—C38	−170.16 (16)
C3—C4—C5—C6	−1.6 (4)	C25—C26—C27—C28	2.4 (3)
C3—C4—S1—C1	26.04 (19)	C25—C26—S2—C23	−25.38 (17)
C4—C3—C8—C7	−1.1 (4)	C26—C25—C30—C29	1.9 (3)
C4—C5—C6—C7	0.0 (4)	C26—C27—C28—C29	0.2 (3)
C5—C4—S1—C1	−157.21 (18)	C27—C26—S2—C23	157.83 (16)
C5—C6—C7—C8	1.0 (5)	C27—C28—C29—C30	−1.8 (4)
C6—C7—C8—C3	−0.5 (4)	C28—C29—C30—C25	0.7 (3)
C8—C3—C4—C5	2.1 (3)	C30—C25—C26—C27	−3.4 (3)
C8—C3—C4—S1	178.83 (17)	C30—C25—C26—S2	179.83 (15)
C9—C1—N1—C2	−76.4 (2)	C31—C23—N2—C24	73.1 (2)
C9—C1—N1—C16	99.6 (2)	C31—C23—N2—C38	−99.91 (19)
C9—C1—S1—C4	78.97 (15)	C31—C23—S2—C26	−76.93 (14)
C9—C10—C11—C12	1.6 (4)	C31—C32—C33—C34	0.4 (3)
C10—C9—C14—C13	−2.7 (4)	C32—C31—C36—C35	1.3 (3)
C10—C11—C12—C13	−2.5 (4)	C32—C33—C34—C35	0.0 (3)
C10—C11—C12—C15	178.7 (2)	C32—C33—C34—C37	179.8 (2)
C11—C12—C13—C14	0.9 (4)	C33—C34—C35—C36	0.3 (3)
C12—C13—C14—C9	1.7 (5)	C34—C35—C36—C31	−0.9 (3)
C14—C9—C10—C11	1.1 (3)	C36—C31—C32—C33	−1.1 (3)
C15—C12—C13—C14	179.7 (3)	C37—C34—C35—C36	−179.51 (19)
C16—C17—C18—C19	−0.4 (3)	C38—C39—C40—C41	−0.5 (3)
C17—C16—C21—C20	2.3 (3)	C39—C38—C43—C42	2.3 (3)
C17—C16—N1—C1	129.4 (2)	C39—C38—N2—C23	44.1 (2)
C17—C16—N1—C2	−54.5 (3)	C39—C38—N2—C24	−129.0 (2)
C17—C18—C19—C20	1.7 (3)	C39—C40—C41—C42	2.8 (4)
C17—C18—C19—C22	−177.7 (2)	C39—C40—C41—C44	−176.1 (2)
C18—C19—C20—C21	−0.9 (4)	C40—C41—C42—C43	−2.6 (3)
C19—C20—C21—C16	−1.1 (4)	C41—C42—C43—C38	0.1 (3)
C21—C16—C17—C18	−1.6 (3)	C43—C38—C39—C40	−2.1 (3)
C21—C16—N1—C1	−48.5 (3)	C43—C38—N2—C23	−133.54 (18)
C21—C16—N1—C2	127.6 (2)	C43—C38—N2—C24	53.3 (2)
C22—C19—C20—C21	178.5 (2)	C44—C41—C42—C43	176.3 (2)
N1—C1—C9—C10	−160.31 (19)	N2—C23—C31—C32	170.50 (16)
N1—C1—C9—C14	18.9 (3)	N2—C23—C31—C36	−8.1 (3)
N1—C1—S1—C4	−50.86 (16)	N2—C23—S2—C26	52.70 (14)
N1—C2—C3—C4	−17.9 (3)	N2—C24—C25—C26	18.3 (3)
N1—C2—C3—C8	169.1 (2)	N2—C24—C25—C30	−169.31 (17)
N1—C16—C17—C18	−179.55 (18)	N2—C38—C39—C40	−179.77 (19)
N1—C16—C21—C20	−179.7 (2)	N2—C38—C43—C42	180.00 (18)
O1—C2—C3—C4	160.1 (2)	O2—C24—C25—C26	−158.83 (19)
O1—C2—C3—C8	−13.0 (3)	O2—C24—C25—C30	13.6 (3)
O1—C2—N1—C1	167.6 (2)	O2—C24—N2—C23	−165.96 (18)
O1—C2—N1—C16	−8.4 (3)	O2—C24—N2—C38	6.9 (3)
S1—C1—C9—C10	72.1 (2)	S2—C23—C31—C32	−62.5 (2)
S1—C1—C9—C14	−108.7 (2)	S2—C23—C31—C36	118.86 (18)
S1—C1—N1—C2	51.4 (2)	S2—C23—N2—C24	−54.8 (2)
S1—C1—N1—C16	−132.69 (16)	S2—C23—N2—C38	132.13 (14)
S1—C4—C5—C6	−178.4 (2)	S2—C26—C27—C28	179.28 (16)

### 2,3-Bis(4-methylphenyl)-2,3-dihydro-4H-1,3-benzothiazin-4-one (2) Hydrogen-bond geometry (Å, º)

Cg4 and Cg8 are the centroids of the C16–C21 and C31–C36 rings, respectively.

**Table:**

*D*—H···*A*	*D*—H	H···*A*	*D*···*A*	*D*—H···*A*
C1—H1···O2	0.98	2.46	3.399 (3)	161
C23—H23···O1^i^	0.98	2.34	3.268 (2)	158
C28—H28···*Cg*8^ii^	0.93	2.60	3.514 (3)	169
C32—H32···*Cg*4	0.93	2.90	3.818 (2)	171

Symmetry codes: (i) *x*, *y*−1, *z*; (ii) *x*+3/2, *y*+1/2, *z*+1.
